# Inner retinal change in a novel rd1-FTL mouse model of retinal degeneration

**DOI:** 10.3389/fncel.2015.00293

**Published:** 2015-07-29

**Authors:** Ursula Greferath, Emily E. Anderson, Andrew I. Jobling, Kirstan A. Vessey, Gemma Martinez, Robb U. de Iongh, Michael Kalloniatis, Erica L. Fletcher

**Affiliations:** ^1^Department of Anatomy and Neuroscience, The University of Melbourne, Melbourne, VICAustralia; ^2^Centre for Eye Health and School of Optometry and Vision Science, University of New South Wales, Sydney, NSWAustralia

**Keywords:** retinitis pigmentosa, Müller cell, *c-fos*, ganglion cell, cone photoreceptor, plasticity

## Abstract

While photoreceptor loss is the most devastating result of inherited retinal degenerations such as retinitis pigmentosa, inner retinal neurons also undergo significant alteration. Detailing these changes has become important as many vision restorative therapies target the remaining neurons. In this study, the rd1-Fos-Tau-LacZ (rd1-FTL) mouse model was used to explore inner retinal change at a late stage of retinal degeneration, after the loss of photoreceptor nuclei. The rd1-FTL model carries a mutation in the phosphodiesterase gene, *Pde6b*, and an axonally targeted transgenic beta galactosidase reporter system under the control of the *c-fos* promoter. Retinae of transgenic rd1-FTL mice and control FTL animals aged 2–12 months were processed for indirect fluorescence immunocytochemistry. At 2 months of age, a time when the majority of photoreceptor nuclei are lost, there was negligible *c-fos* reporter (FTL) expression, however, from 4 months, reporter expression was observed to increase within subpopulations of amacrine and ganglion cells within the central retina. These areas of inner retinal FTL expression coincided with regions that contained aberrant Müller cells. Specifically, these cells exhibited reduced glutamine synthetase and Kir4.1 immunolabelling, whilst showing evidence of proliferative gliosis (increased cyclinD1 and glial fibrillary acidic protein expression). These changes were limited to distinct regions where cone photoreceptor terminals were absent. Overall, these results highlight that distinct areas of the rd1-FTL central retina undergo significant glial alterations after cone photoreceptor loss. These areas coincide with up-regulation of the *c-fos* reporter in the inner retina, which may represent a change in neuronal function/plasticity. The rd1-FTL mouse is a useful model system to probe changes that occur in the inner retina at later stages of retinal degeneration.

## Introduction

Over recent years there has been a surge of interest in vision restoring therapies that replace the function of dead or dying photoreceptors using a variety of genetic, electronic or transplantation methods (Busskamp et al., 2008; O’Brien et al., 2012). For any of these treatments to succeed, a basic requirement is that the circuitry of the inner retina remains functionally intact and capable of passing visual information to higher visual centers. Yet, over recent years, the notion that the inner retina remains intact following complete loss of photoreceptors has been questioned (Jones et al., 2003; Marc et al., 2003, 2007; Strettoi et al., 2003; Jones and Marc, 2005; Chua et al., 2009). A range of changes occur in both neurons and glial cells in animal models of inherited retinal degeneration, as well as humans with retinitis pigmentosa (RP). These changes are classified into three phases based on retinal anatomy (Jones and Marc, 2005); Phases 1 and 2 refer to the periods of rod photoreceptor and cone photoreceptor death, respectively, while phase 3 is characterized by major anatomical changes in the inner retina including re-wiring of neural processes and the formation of a glial seal that encases the remaining retina (Marc et al., 2003). As most vision restorative therapies will be employed after photoreceptor death, a greater understanding of the events that occur in phase 3 degeneration will assist in optimizing these therapeutic approaches.

The rd1 mouse is the most widely studied mouse model of retinal degeneration (Farber, 1995; Fletcher et al., 2011). It carries a gene mutation in the β subunit of phosphodiesterase 6 (*Pde6b*), which is a similar genetic defect to patients with autosomal recessive RP (Pittler and Baehr, 1991; McLaughlin et al., 1993). Studies have documented both functional and anatomical changes in this mouse model of degeneration (Strettoi and Pignatelli, 2000; Strettoi et al., 2002; Jones et al., 2003; Margolis et al., 2008). Apart from the obvious structural changes in the outer retina (the loss of photoreceptors), there are also functional changes in bipolar and ganglion cells within this early period of degeneration (Stasheff, 2008; Chua et al., 2009; Stasheff et al., 2011; Gibson et al., 2013). However, at later stages of degeneration after photoreceptor loss, neurons of the inner retina also show significant anatomical alterations, including migration of somata to ectopic sites, the establishment of aberrant synapses, neuronal death, and glial remodeling (Jones et al., 2003; Marc et al., 2003; Chua et al., 2013). Finally, while neurochemical changes have been described both at early and late stages of degeneration in the rd1 mouse, as well as in other models of retinal degeneration (Fletcher and Kalloniatis, 1996, 1997; Marc et al., 2003; Jones and Marc, 2005; Gibson et al., 2013), the underlying cause and relationship between the different forms of inner retinal change is not clear. For that reason a model system probing these inner retinal changes would be of use.

The Fos-Tau-LacZ (FTL) mouse has an axonally targeted reporter system under the regulation of the *c-fos* promoter (Wilson et al., 2002). *C-fos* is an intermediate early gene that is known to be up-regulated in neurons in response to neural activity, cell death, and/or plasticity (Marti et al., 1994; Rich et al., 1997; Wilson et al., 2002; Greferath et al., 2004, 2009). FTL mice have been useful for visualizing neural circuits activated by a range of stimuli, including those involved in regulating water balance (Wilson et al., 2002), learning and memory (Ali et al., 2009, 2012) and respiratory control within the nuclei of the brainstem (Niblock et al., 2012). When FTL mice are bred with rd1 mice, photoreceptors display increased *c-fos* reporter labeling from an early age that follows closely the time course of photoreceptor death (Greferath et al., 2009). However, nothing is known about *c-fos* reporter (FTL) regulation at later stages of degeneration in the rd1-FTL mouse and whether this relates to inner retinal neural change.

In view of the increase in aberrant neural activity, plasticity changes and possible cell death that takes place within the inner retina at late stages of degeneration, the rd1-FTL mouse could provide a useful tool for studying the cellular changes that occur in the inner retina well after photoreceptor cell death. In this study we examined the time course of *c-fos* reporter expression in the rd1-FTL mouse and characterized the accompanying glial and neuronal alterations. Our results show that *c-fos* reporter (FTL) expression is upregulated in inner retinal neurons within discrete patches in the central retina from 4 months of age. Moreover, increased FTL expression coincides with regions displaying considerable Müller cell change and loss of cone photoreceptor terminals. These areas may represent regions of the retina from which the glial seal may form. Targeting restorative therapies distant to these retinal areas may improve outcomes.

## Materials and Methods

### Animals

All animal procedures were performed in accordance with the University of Melbourne Animal Experimentation Ethics Committee and with guidelines outlined by the National Health and Medical Research Council and the ARVO Statement for the Use of Animals in Ophthalmic and Vision Research.

Rd1-FTL double-mutant mice were created by breeding rd1 homozygous mice with heterozygous FTL transgenic animals (Wilson et al., 2002). Rd1 mice were originally obtained from Professor Debora Farber, University of California, Los Angeles, CA, USA and backcrossed more than 10 times with the C57Bl6J^ARC^ obtained from the Animal Resource Centre (Western Australia). FTL mice were originally generated at the University of Melbourne (Parkville, Australia; Wilson et al., 2002). The rd1-FTL mouse expresses a mutation in the β subunit of *Pde6b* gene, resulting in retinal degeneration, together with an axonally targeted transgenic marker gene (β-galactosidase) that labels all neurons that have *c-fos* promoter activation (Greferath et al., 2009). Mice examined in this study were aged from 2 to 12 months and control heterozygous FTL mice were age matched for comparison (at least *n* > 7 rd1-FTL and FTL at each age group per outcome measure). All mice used in this study were confirmed to be free of the rd8 mutation in the *Crb1* gene (Mattapallil et al., 2012).

### Tissue Collection and Fixation

Mice were killed by an overdose of sodium pentobarbital (Merial Australia, Parramatta, Australia: 120 mg/kg, intraperitoneal). Eyes were then immediately dissected, the anterior segment and lens discarded and the posterior eyecup fixed in one of two ways depending on the type of immunofluorescence to be performed. Posterior eye cups that were processed for indirect immunofluorescence were fixed in 4% paraformaldehyde (PFA) in 0.1 M phosphate buffer, pH 7.4 (phosphate buffered, PB) for 30 min, washed in PB and subsequently processed through graded sucrose solutions (10, 20, and 30% w/v sucrose in PB). Samples were stored at –80°C until use.

A subset of eyes from rd1-FTL and FTL mice (*n* > 8 eyes) were processed for β-galactosidase histochemistry, whereby the mouse was transcardially perfused with 12 ml of 5% sucrose in dH_2_O, followed by 12 ml of PFA with 0.005% glutaraldehyde in PB. Eyes were immediately dissected and a hole pierced in the eyecup at the corneal margin with a 30G needle. Eyes were then placed in 4% PFA and the anterior eyecup and lens were removed. The posterior eyecup was postfixed in 4% PFA for 10 min and then placed in phosphate buffered saline (PBS). All eyes were immediately processed for enzyme histochemistry.

### β-Galactosidase Histochemistry on Wholemount Retinae

Retinae were dissected from the posterior eyecup and placed in the staining buffer (5 mM magnesium chloride, 5 mM potassium ferrocyanide, 5 mM potassium ferricyanide, and 0.4 mg/ml 5-Bromo-4-chloro-3-indolyl-B-D-galatocpyranoside) for 16 h under rotation at room temperature (Greferath et al., 2004, 2009). To stop the reaction, an equivalent volume of 4% PFA was added to the solution and the retina was washed in PBS and mounted onto 0.5% gelatine/5% chromium potassium sulfate-coated slides. Wholemounts were air-dried on slides and dehydrated through graded alcohols to histolene and mounted in Safety Mount (Fronine, Riverstone, Australia). Wholemount retinae processed for β-galactosidase histochemistry were viewed using a light microscope with either a 4 × air objective or 40 × oil objective and tissue was imaged using an ImagePoint cooled CCD camera (Photometrics LTD, Tucson, AZ, USA) and assessed using Windows imaging software (Digital Optics Ltd., Auckland, NZ). The extent of β-galactosidase expression (FTL labeling) was quantified as a percentage of total retinal area from *n* ≥ 8 retinae (mm^2^ FTL labeling/mm^2^ retinal area).

### Immunofluorescence

Changes in cells in FTL and rd1-FTL mice were evaluated on vertical sections of retina using indirect immunofluorescence as previously described (Fletcher et al., 2000; Downie et al., 2010; Vessey and Fletcher, 2012). Briefly, sections (14 μm in thickness) were cut from OCT (Tissue Tek, Sakura Finetek, Tokyo, Japan) embedded eyecup blocks and mounted onto 0.5% gelatine/5% chromium potassium sulfate coated slides that were then air-dried. Slides were washed in PB and primary antibody was applied overnight. Following incubation, sections were again washed in PB and secondary antibody was applied for 40 min. **Table 1** contains a list of the primary and secondary antibodies that were used. All antibodies were diluted in buffer consisting of 3% normal goat serum, 1% bovine serum albumin and 0.5% Triton X-100 in PB. Cell death was measured using a commercially available fluorometric terminal dUTP nick-end labeling (TUNEL) kit (DeadEnd Fluoro metric TUNEL system, Promega, Madison, WI, USA). Slides were then washed in PB, and mounted and cover-slipped with fluorescent mounting medium (DAKO, Carpinteria, CA, USA).

**Table 1:** List of the primary and secondary antisera used in this study.

Antiserum	Dilution/Species	Source/Cat. No.
β-galactosidase	1:10,000 Rabbit polyclonal	MP Biomedicals; Cat#08559761
β-galactosidase	1:10,000 Chicken polyclonal	Abcam; Cat#AB9361
Calretinin	1:500 Mouse monoclonal	Swant; clone 6B3
CyclinD1	1:1 Rabbit polyclonal	Merck Millipore; Cat#04-1151
GABA	1:500 Guinea pig polyclonal	Merck Millipore; Cat#AB175
Glutamate	1:45,000 Rabbit polyclonal	Gift from Dr Robert E Marc, available through Chemicon International; Cat #AB133
Glial fibrillary acid protein	1:20,000 Rabbit polyclonal	DAKO; Cat# Z0334
Glycine	1:50,00 Rabbit polyclonal	Gift from Prof David Pow, University of Queensland, Brisbane, QLD, Australia
GLAST (EAAT1)	1:1,000 Rabbit polyclonal	Abcam; Cat# AB41751
Vesicular glutamate transporter 1	1:20,000 Guinea pig, polyclonal	Merck Millipore; Cat #AB5905
Glutamine synthetase	1:1,000 Mouse monoclonal	Merck Millipore; Cat# MAB302
Tyrosine hydroxylase	1:1,000 Mouse monoclonal	Chemicon International; Cat #MAB318
Kir4.1	1:1,000 Rabbit polyclonal	Merck Millipore; Cat#AB5818
Neuron specific nuclear protein	1:500 Mouse monoclonal	Merck Millipore; Cat# MAB377
Peanut Agglutinin-Rhodamine	1:250 Fluorescent Lectin	Vector Labs; Cat #RL-1072
Sox9	1:500 Rabbit polyclonal	Merck Millipore; Cat# AB5535
Anti-Rabbit Alexa594	1:800 Goat polyclonal	Life Technologies; Alexa Fluor^®^ Cat# A11037
Anti-Mouse Alexa488	1:500 Goat polyclonal	Life Technologies; Alexa Fluor^®^_Cat# A11001
Anti-Guinea pig Alexa594	1:500 Goat polyclonal	Life Technologies; Alexa Fluor^®^_Cat# A11076
Anti-Guinea pig Alexa488	1:500 Goat polyclonal	Life Technologies; Alexa Fluor^®^ Cat# A11073
Anti-Chicken Alexa594	1:500 Goat polyclonal	Life Technologies; Alexa Fluor^®^ Cat# A11042
Anti-Mouse Alexa647	1:500 Goat polyclonal	Life Technologies; Alexa Fluor^®^ Cat# A21235
BisBenzimide H	0.1 mg/mL	Sigma Aldrich; Cat#14530

Retinal sections processed for immunofluorescence and TUNEL were viewed on a confocal laser scanning microscope (LSM 510 PASCAL, Zeiss, Oberkochen, Germany) using either a 20 × air objective or a 40 × oil objective and images were captured at a resolution of 512 × 512 or 1024 × 1024 pixels using Zeiss LSM image browser software and an appropriate fluorescence filter (Alexa TM 594/CY3: excitation 568 nm, emission filter 605/32; Alexa TM 488/FITC: excitation 488 nm, emission filter 522/32; Alexa TM 647/CY5: excitation 647 nm, emission filter 680/32). Images were collated using Adobe Photoshop CS5 (Adobe Systems, San Jose, CA, USA).

### Histological Analysis

The immunolabeling of each antibody was assessed across at least *n* ≥ 6 eyes, with three separate sections imaged per eye. The co-localisation of FTL labeling with specific cell markers was quantified and expressed as a percentage of the total number of FTL positive cells [%, (co-label of FTL and cell marker/total cells stained for FTL) × 100]. The markers of retinal Müller cells were quantified in control (FTL) and rd1-FTL retinae (areas exhibiting FTL labeling and neighboring areas lacking expression of FTL) as cell number per mm of retina (*n* ≥ 6). Total and inner retinal thickness measures were calculated from central retinal sections (*n* = 6 eyes, at least three sections per eye) that showed FTL labeling and neighboring regions that lacked FTL expression (assessed after indirect immunofluorescence, processed with ImageJ 1.43 freeware, NIH).

### Statistical Analysis

A one-way analysis of variance (ANOVA) with a Tukey’s multiple comparison *post hoc* test was used to statistically compare the change in *c-fos* reporter labeling across the rd1 retina. The quantification of glutamine synthetase (GS), Sox9, and cyclin D1 immunolabeling was analyzed using a two-way ANOVA, with Tukey’s multiple comparison *post hoc* test to evaluate the differences. GraphPad Prism (San Diego, CA, USA) was used to graph data and to perform the above statistical analyses.

## Results

This study examined the retinal changes that take place at late stages of degeneration (6–7 month-old animals) in the rd1-FTL mouse model of retinal degeneration. The rd1-FTL mouse has an axonally targeted reporter system under the control of the *c-fos* promoter (Wilson et al., 2002), as well as carrying a homozygous mutation in *Pde6b*. In the results described below, an increase in the *c-fos* reporter is referred to as “FTL” labeling.

### FTL Labeling is Increased in Inner Retinal Neurons Well after Photoreceptor Degeneration

**FIGURE 1:**
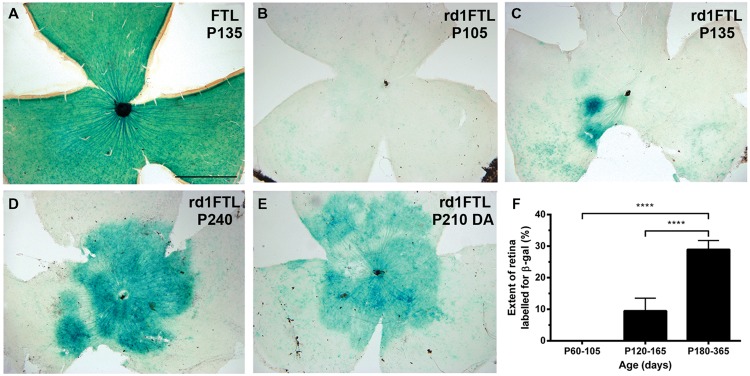
**Fos-Tau-LacZ (FTL) reporter expression increases in the rd1-FTL retina as a function of age.** Flatmount retinae from control FTL (**A**, P135), rd1-FTL (**B**, P105; **C**, P135; **D**, P240; **E**, dark adapted rd1-FTL aged P210) mice were processed for FTL expression using β-galactosidase histochemistry. In contrast to the wild-type retina that showed FTL labeling throughout the retina **(A)**, the rd1-FTL aged P105 **(B)** showed no FTL labeling. However, at P135 **(C)** rd1-FTL retina showed distinct labeling near the optic nerve, that subsequently expanded across the central retina **(D**,**E)**. The expression of the FTL reporter as a function of retinal area was quantified at different ages from P105 to P365 **(F)**. Data presented as mean ± SEM, *n* ≥ 8 per group, ^∗∗∗∗^*p* < 0.0001 (one-way ANOVA). Scale bar for **(A**–**E)** = 1 mm.

Using β-galactosidase histochemistry, the time course of FTL labeling was assessed in rd1-FTL mice up until 1 year of age and compared to age-matched control FTL mice. In the adult (post-natal day 135, P135) control retina (**Figure 1A**), FTL labeling covered the entire retina and as described previously, was found in a range of inner retinal neurons (Greferath et al., 2004). By contrast, FTL labeling was virtually absent in the rd1-FTL retina at a similar age (P105, **Figure 1B**). By P135 (**Figure 1C**), there were discrete regions of FTL labeling lateral to the optic disk in the rd1-FTL retinae. Over time, this labeling spread to cover the central retina (**Figure 1D**), although the peripheral retina never showed labeling, even after 12 months (data not shown).

Previous studies in young rd1-FTL animals (≤P30) have shown that FTL labeling in the retina is influenced by light activation (Greferath et al., 2004). In order to determine whether this increase in FTL labeling observed at this late stage of degeneration (≥P135) was also dependent on light activation, 7 month-old rd1-FTL animals were dark-adapted prior to enucleation and β-galactosidase histochemistry. No difference in the labeling pattern between the light (**Figure 1D**) and dark-adapted (**Figure 1E**) rd1-FTL retinae was observed, indicating this increase in FTL labeling is independent of light-induced activation. Quantifying the extent of FTL labeling in the retina showed an age-dependent increase in expression after 105 days post-natal, with 29 ± 3% of the total retinal area exhibiting FTL labeling out to 1 year of age (**Figure 1F**, one-way ANOVA, *p* < 0.0001, *n* ≥ 8 per group).

**FIGURE 2:**
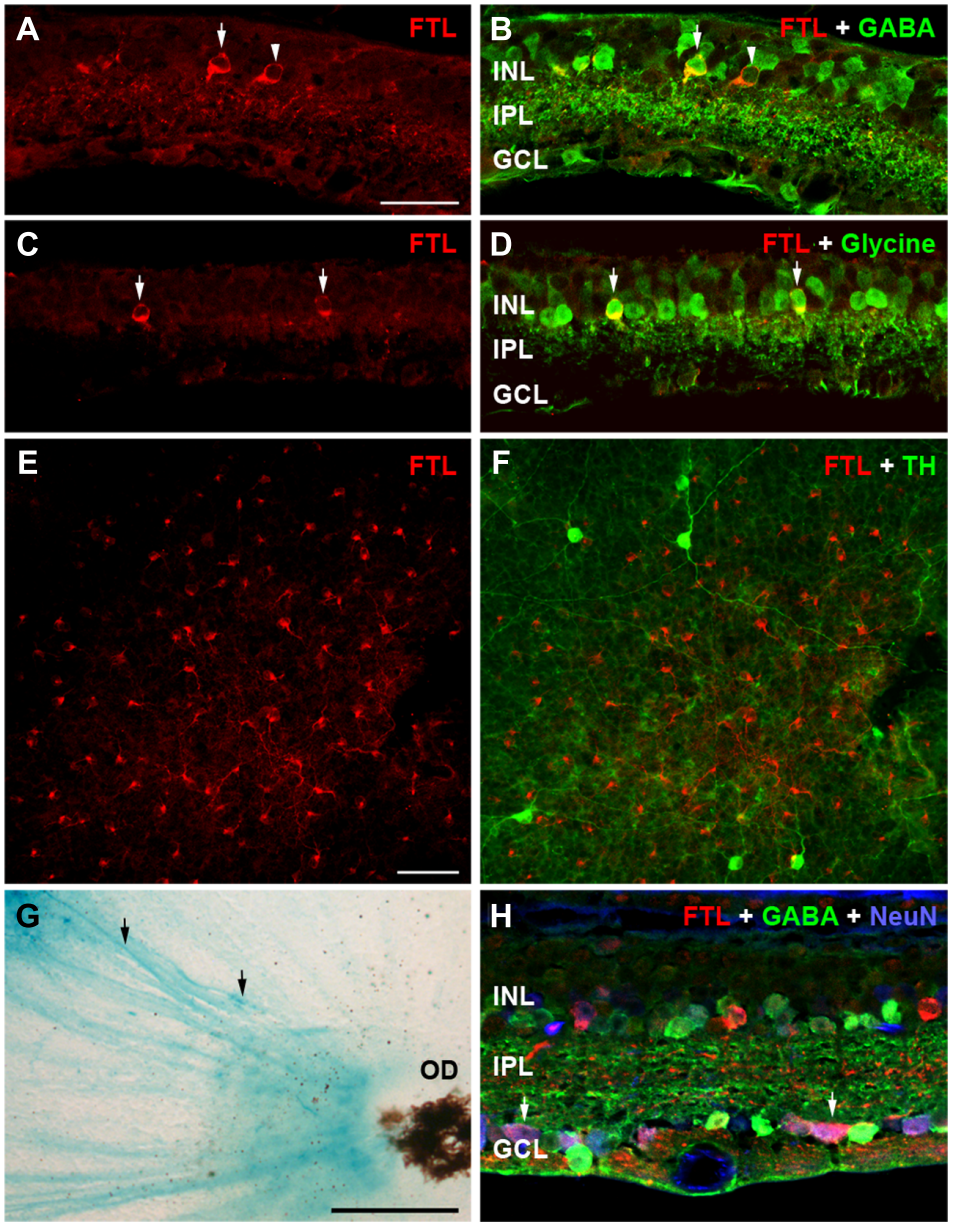
**Amacrine and ganglion cells express the FTL reporter within the inner retina of rd1-FTL.** Retinal sections of rd1-FTL mouse (6 months of age) were labeled with anti-β-galactosidase (**A**,**C**,**E**; red), which identifies FTL reporter expression (see white arrows in **A**,**C**). Retinal sections were co-labeled with GABA **(B)**, or glycine **(D)** showing FTL was co-localized within subpopulations of GABA immunoreactive (**B**, white arrows) and glycine immunoreactive (**D**, white arrows) amacrine cells. Retinal wholemounts were double labeled with anti-β-galactosidase and tyrosine hydroxylase (**E**,**F**; respectively) and no co-localization was evident. Rd1-FTL wholemount from a 4.5 month-old animal was also labeled using β-galactosidase histochemistry showing ganglion cell axons projecting to the optic nerve express the reporter (**G**, black arrows). This ganglion cell expression was confirmed in vertical sections of rd1-FTL retina, which were triple labeled for β-galactosidase (red), GABA (green) and NeuN (Blue). There are numerous cells in the GCL that are co-labeled with FTL and NeuN (**H**, white arrows). Abbreviations: INL, inner nuclear layer; IPL, inner plexiform layer; GCL, ganglion cell layer, OD, optic disk. Scale bar for **(A**–**F**,**H)**: 50 μm; **(G)**: 250 μm.

The retinal cell types that were responsible for this increase in FTL expression were evaluated using immunofluorescence in 6 month-old rd1-FTL retinae. As shown in **Figures 2A,C** and **E**, cells in the inner retina were positive for FTL labeling (anti-β-Gal, red). In order to determine the types of cells expressing FTL immunoreactivity, double labeling experiments were performed with markers of amacrine cells including, GABA, glycine, and tyrosine hydroxylase. FTL was observed to co-localize with GABA (**Figure 2B**, arrows) and glycine (**Figure 2D**, arrows), but not tyrosine hydroxylase (**Figure 2F**), suggesting that in late stage degeneration, GABAergic and glycinergic amacrine cells express the *c-fos* reporter, while dopaminergic amacrine cells do not. This is unlike the inner retinal labeling observed in this model during the early stages of photoreceptor degeneration (<P26) where dopaminergic amacrine cells also express the *c-fos* reporter (Greferath et al., 2009).

β-galactosidase histochemical labeling of the rd1-FTL retina showed evidence of reporter expression within ganglion cell axons (**Figure 2G**, black arrows). In order to assess ganglion cell expression of the *c-fos* reporter, NeuN was used to label all neurons in the ganglion cell layer (GCL), while GABA immunolabeling was used to exclude displaced amacrine cells. Triple labeling with antibodies to β-Gal, NeuN, and GABA identified numerous ganglion cells that were labeled for FTL (NeuN positive, GABA negative, **Figure 2H**, see arrows). Quantification of FTL positive cells in the inner retina revealed that 28 ± 8% of FTL immunoreactive cells were strongly GABA immunoreactive, 41 ± 20% were strongly glycine immunoreactive, while within the GCL, 16 ± 5% were NeuN^+^/GABA^-^ neurons (*n* ≥ 6 animals). These data suggest that specific subpopulations of conventional amacrine cells and ganglion cells express the *c-fos* reporter during the later stages of retinal degeneration.

### FTL expression during Late-Stage Degeneration Does Not Reflect Inner Retinal Thinning, Nor Exclusively Correlate with Remodeling

**FIGURE 3:**
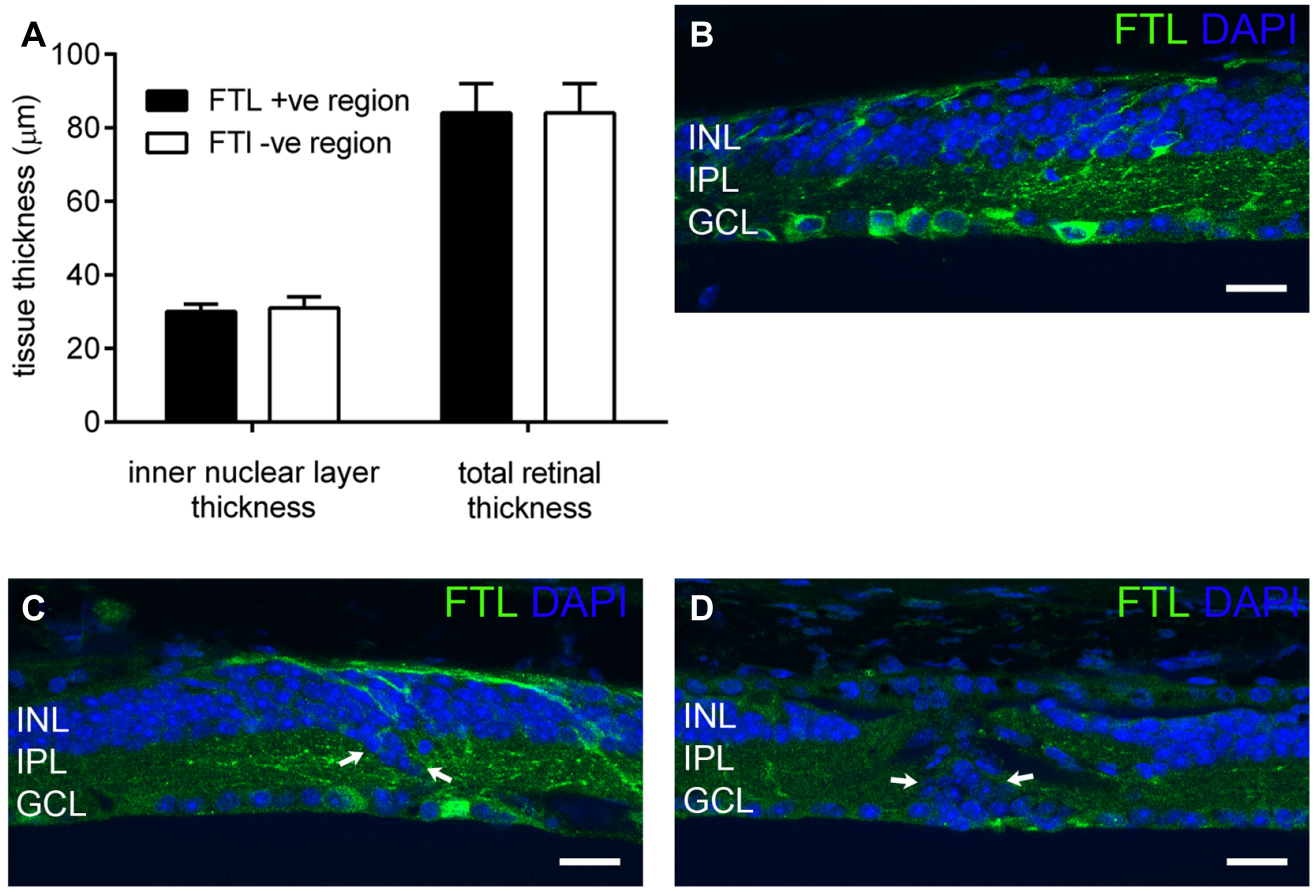
**Fos-Tau-LacZ reporter expression in the rd1-FTL retina is not due to cell death nor correlated with remodeled inner retina.** Total and inner nuclear layer thickness measures were measured from vertical retinal sections in areas exhibiting FTL expression, as well as neighboring areas showing no FTL expression **(A)**. Additionally, vertical sections of rd1-FTL retina were labeled for FTL (anti-β-galactosidase, green) and DAPI (blue). Inner retinal layer and total retinal thickness’ were similar between FTL^+^ sand FTL^-^ regions **(A)**. Increased FTL reporter expression was observed in inner retinal regions where the structure remained intact **(B)**, as well as in regions where there was evidence of anatomical remodeling (arrows, **C**). Furthermore, other regions that exhibited DAPI labeled nuclei which were mislocated within the IPL, were FTL negative (arrows, **D**). Data presented as mean ± SEM, *n* ≥ 6 per group, *p* > 0.05 (two-way ANOVA). Abbreviations: INL, inner nuclear layer; IPL, inner plexiform layer; GCL, ganglion cell layer. Scale bar: 50 μm.

*c-fos* is known to be upregulated during cell death (Marti et al., 1994). Indeed, our previous study of rd1-FTL mice showed FTL labeling in photoreceptor nuclei during the period of rod loss between post-natal day 10 and 30 (Greferath et al., 2009). In order to determine whether the amacrine and ganglion cell labeling observed at late stages of retinal degeneration was associated with cell death, inner nuclear layer (INL) thickness was quantified in FTL immunolabeled regions and non-labeled areas that were immediately adjacent. The INLs of the FTL positive regions were not significantly different to the adjacent negative regions (**Figure 3A**, FTL^+^ 30 ± 2, FTL^-^ 31 ± 3 μm, *p* > 0.05, *n* ≥ 6 animals), while total retinal thickness was also unchanged (**Figure 3A**, FTL^+^ 84 ± 8, FTL^-^ 84 ± 8 μm, *p* > 0.05, *n* ≥ 6 animals). To confirm the lack of cell death involvement in FTL labelling, TUNEL labeling was performed within the inner retina of 4 and 6 month-old rd1-FTL mice. Limited staining was detected, similar to that observed in age-matched FTL controls (data not shown). These findings suggest that at this late stage of retinal degeneration, the FTL labeling observed in the amacrine and ganglion cells of the rd1-FTL retina is unlikely to be the result of cell death.

As *c-fos* expression has been associated with neural plasticity, retinal areas exhibiting anatomical remodeling were correlated to FTL labeling. As shown in **Figure 3B**, FTL labeling occurred in areas with normal inner retinal morphology, as well as in regions with abnormally localized nuclei in the IPL, indicative of anatomical remodeling (arrows in **Figure 3C**). However, other regions that exhibited mislocalised DAPI-labeled nuclei within the IPL, were FTL negative (arrows in **Figure 3D**). Thus, FTL labeling in the inner retina did not exclusively coincide with areas that showed evidence of remodeling, suggesting that FTL up-regulation does not solely reflect neuronal plasticity changes in the rd1-FTL retina.

### FTL Labeling of Inner Retinal Neurons is Localized to Regions of Aberrant Glia

**FIGURE 4:**
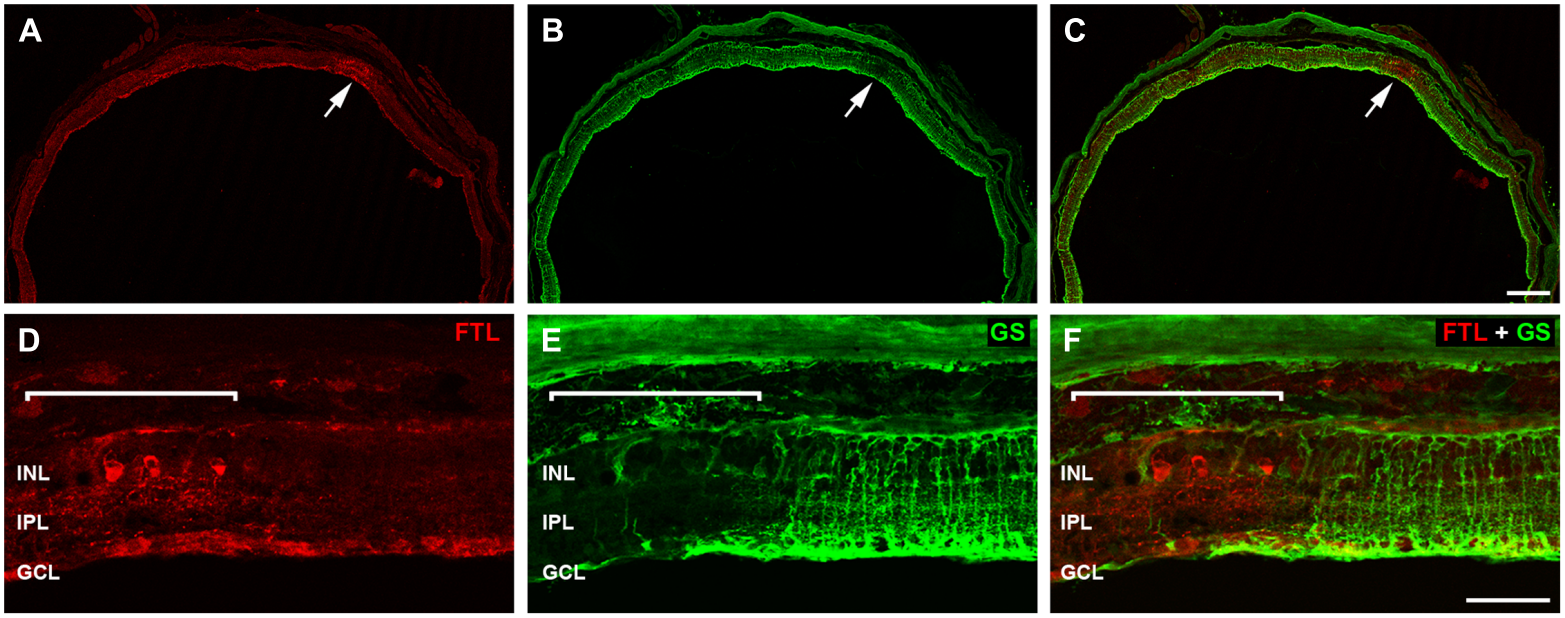
**Fos-Tau-LacZ positive regions within the rd1-FTL retina show Müller cell alterations.** Vertical sections of the rd1-FTL mouse retina (6 month-old) were labeled for FTL (**A**,**D**; anti-β-galactosidase, red) and glutamine synthetase (GS; **B**,**E**; green) and areas of co-localisation assessed **(C**,**F)**. Regions that are intensely labeled for FTL correspond to regions that show low GS labeling (white arrow; **B, C**). High magnification of the FTL expressing region within the rd1-FTL retina is indicated by the white bracket and corresponds to an area of low GS labeling. Abbreviations: INL, inner nuclear layer; IPL, inner plexiform layer; GCL, ganglion cell layer. Scale bar: 250 μm **(A**–**C)**, 50 μm **(D**–**F)**.

The labeling within the rd1-FTL retina was patchy, with some regions showing FTL in cell somata of the INL (**Figure 4A** arrow, **Figure 4D** highlighted region), whereas neighboring areas showed no cell soma labeling. There was, however, a relatively consistent labeling within the GCL likely due to *c-fos* reporter expression within axons (**Figure 4D**, also see **Figure 2G**). Müller cell labeling with GS (green; Bringmann et al., 2009) also showed a variability in staining (**Figure 4B** arrow, **Figure 4E** highlighted region) and revealed an intriguing inverse correlation, with FTL expression observed in retinal regions that were either devoid of, or showed a major reduction in, GS labeling (**Figures 4C,F**).

**FIGURE 5:**
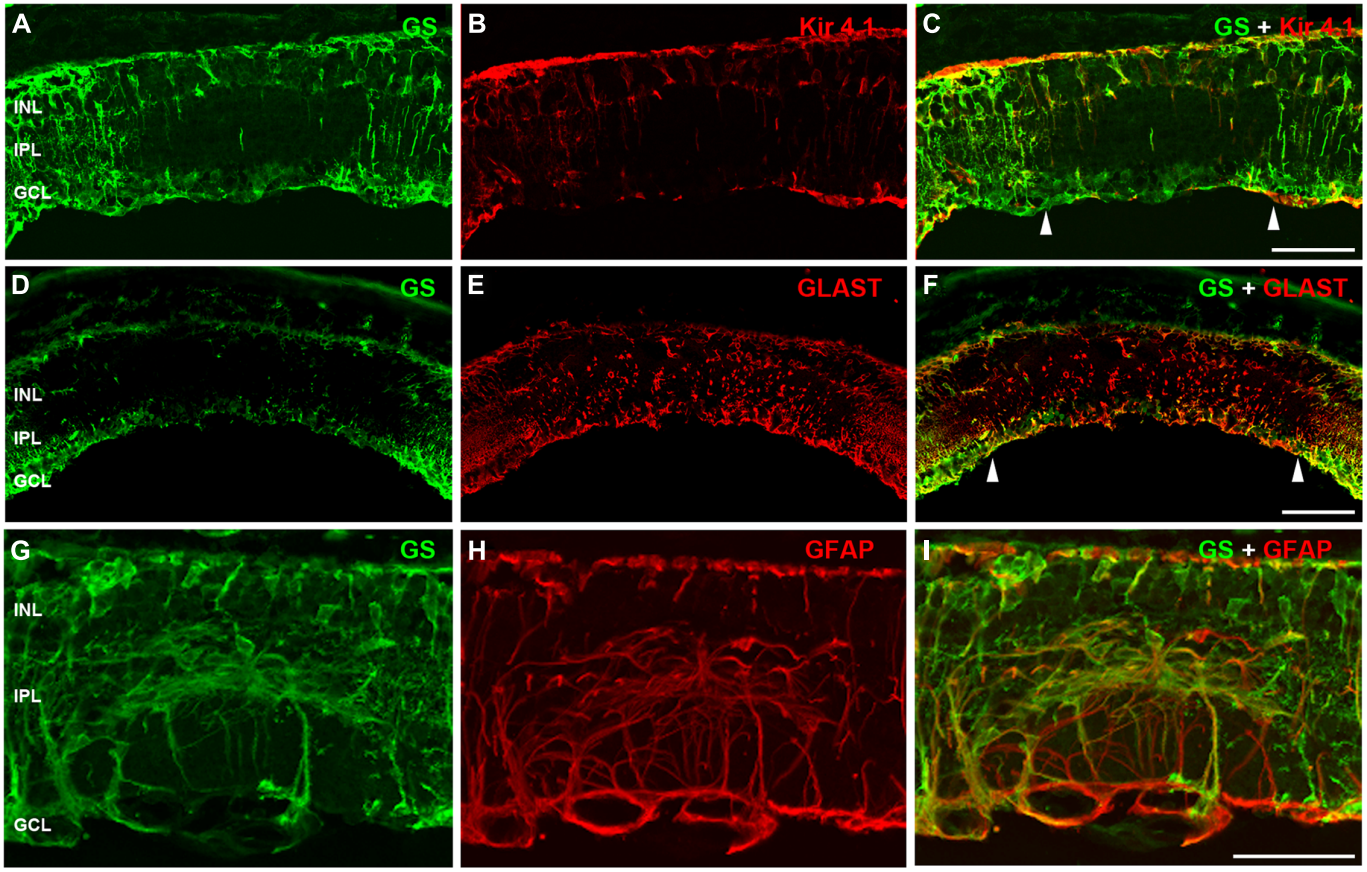
**Müller cells are altered in the FTL positive regions within the rd1-FTL retina.** Vertical sections of rd1-FTL retinae (7 month-old) were labeled for glutamine synthetise **(A,D,G)**, Kir4.1 **(B)**, GLAST **(E)**, or GFAP **(H)**, with the double label shown in **(C,F,I)**. Areas that showed little GS labeling (in between arrow heads in **C** and **F**) corresponded to Müller cells that lacked Kir4.1. Despite this, these areas still showed GLAST immunolabelling. These altered regions were also positive for the gliosis marker GFAP. Abbreviations: INL, inner nuclear layer; IPL, inner plexiform layer; GCL, ganglion cell layer. Scale bar: 50 μm.

Müller cells are integral to maintenance of retinal structure and function, being involved in potassium siphoning, glutamate and GABA turnover, chromophore recycling, energy metabolism and maintenance of the blood retinal barrier (Bringmann et al., 2006). To examine whether these Müller cell metabolic processes might be compromised, rd1-FTL retinae were labeled for Kir4.1 the predominant glial inwardly rectifying potassium channel (Kofuji et al., 2000), GLAST, the principal glutamate transporter expressed by Müller cells (Lehre et al., 1997; Rauen et al., 1998), and glial fibrillary acidic protein (GFAP), a marker of Müller cell gliosis. As shown in **Figure 5**, regions of the rd1-FTL retina that showed reduced GS immunolabeling also showed reduced Kir4.1 staining (**Figures 5A–C**). However, while GLAST-labeling was still detected within these regions (**Figures 5D–F**), it appeared abnormal when compared to neighboring GS-expressing regions. These altered regions showed extensive GFAP labeling (**Figures 5G–I**), which has been reported previously in the rd1 mouse retina (Stasheff, 2008; Chua et al., 2009; Stasheff et al., 2011; Gibson et al., 2013). These data suggest that while GS and Kir4.1 expression is significantly reduced, gliotic Müller cells with the potential for glutamate uptake remained within these aberrant regions.

**FIGURE 6:**
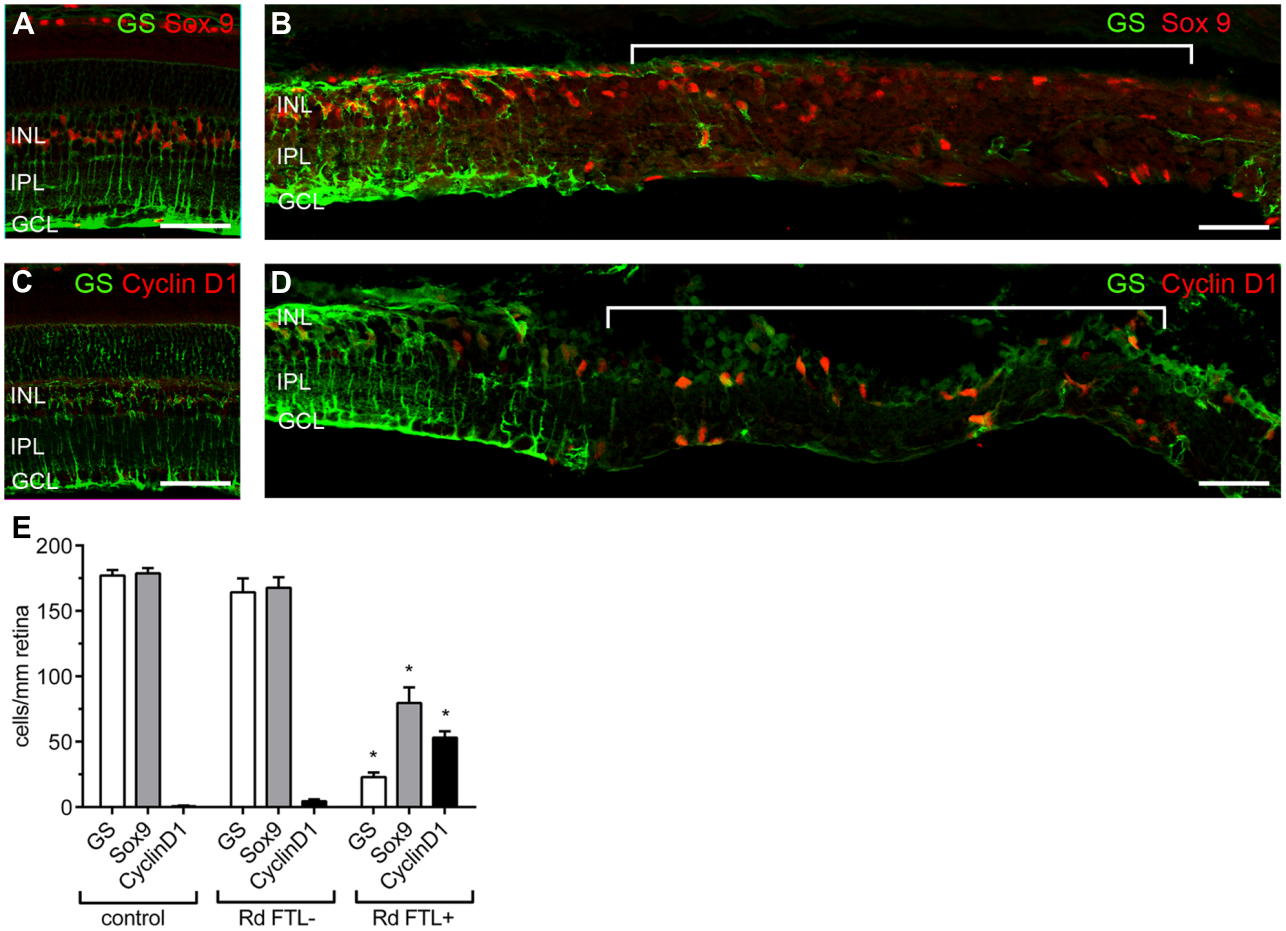
**Müller cells in the FTL positive regions within the rd1-FTL retina show evidence of proliferative gliosis.** Vertical sections of 6 month-old control FTL **(A)** and rd1-FTL **(B)** retina were double-labeled for GS (green) and SOX9 (red). Sox 9 immunolabeled Müller cell somata were present in regions that showed little or no labeling for GS. Control FTL **(C)** and rd1-FTL **(D)** were double labeled for GS (green) and Cyclin D1 (red). Cyclin D1 expression was only found in regions that showed little or no GS. GS, Sox9, and ClyclinD1 expression was quantified in control FTL retinae, and in rd1-FTL retinae in areas with and without FTL reporter expression **(E)**. Data are expressed as mean immunoreactive cells per mm retina ±SEM *n* ≥ 6 per group, ^∗^*p* < 0.0001 (two-way ANOVA). Abbreviations: INL, inner nuclear layer; IPL, inner plexiform layer; GCL, ganglion cell layer. Scale bar: 50 μm.

These results imply that after photoreceptor degeneration in the rd1-FTL retina, localized subpopulations of amacrine and ganglion cells show increased FTL expression in regions where Müller cells are dysfunctional or absent. In order to determine if Müller cell loss occurred in these FTL-expressing regions, retinal sections were co-labeled for GS and Sox9, a Müller cell nuclear marker. As shown in **Figure 6**, Sox 9 labeling was 100% co-localized with GS positive Müller cells in control retinae (**Figure 6A**), and in areas of the rd1-FTL retina that were GS positive (**Figure 6B**). By contrast, FTL positive areas that almost completely lacked GS, still showed some cells that were Sox9 immunoreactive (**Figure 6B**, highlighted region). Quantifying the number of GS and Sox9 immunoreactive Müller cells in control FTL and rd1-FTL (FTL^+^ or FTL^-^ regions) retinae (**Figure 6E**), showed a significant reduction in immunoreactive somata between FTL positive areas and control retinae (GS, –87 ± 2%; Sox9 –56 ± 7%; two-way ANOVA, *p* < 0.0001, *n* ≥ 6) as well as FTL positive and negative regions (GS, –86 ± 2%; Sox9 –53 ± 7%; two-way ANOVA, *p* < 0.0001, *n* ≥ 6). There were no significant differences between control retinae and FTL negative regions of the rd1-FTL retinae (GS, –7 ± 6%; Sox9, –6 ± 5%: two-way ANOVA, *p* > 0.05, *n* ≥ 6).

As these FTL-positive areas exhibited several markers of proliferative gliosis (reduced GS, and Kir4.1 expression and increased GFAP), cyclinD1 was used to determine whether Müller cells in these affected regions had altered cell cycle regulation. Increased cyclinD1 expression in Müller cells has been previously reported during late stage retinal degeneration (Vessey et al., 2014). As shown in **Figure 6C**, no cells in the age-matched control retinae showed cyclinD1 expression. However, within FTL^+^/GS^-^ regions of the rd1-FTL retina, numerous Müller cells showed cyclinD1 expression (**Figure 6D**, highlighted region). When quantified (**Figure 6E**), a significantly higher number of cyclinD1 immunoreactive Müller cells were found in regions of FTL labeling compared to control (FTL^+^ regions 53 ± 5 cells/mm retina; control FTL 0.6 ± 0.4 cells/mm retina; two-way ANOVA, *p* < 0.0001, *n* ≥ 6), or neighboring FTL negative regions (FTL^+^ regions 53 ± 5 cells/mm retina; FTL^-^ regions 4 ± 2 cells/mm retina; two-way ANOVA, *p* < 0.0001, *n* ≥ 6). These results highlight that well after photoreceptor degeneration, inner retinal FTL expression coincides with distinct regions that show reduced Müller cell number (reduced Sox9), with the remaining cells exhibiting aberrant expression of functional markers, including Kir4.1, GS, and cyclin D1.

### Cone Loss Occurs in Regions of Glial Loss and Abnormality

**FIGURE 7:**
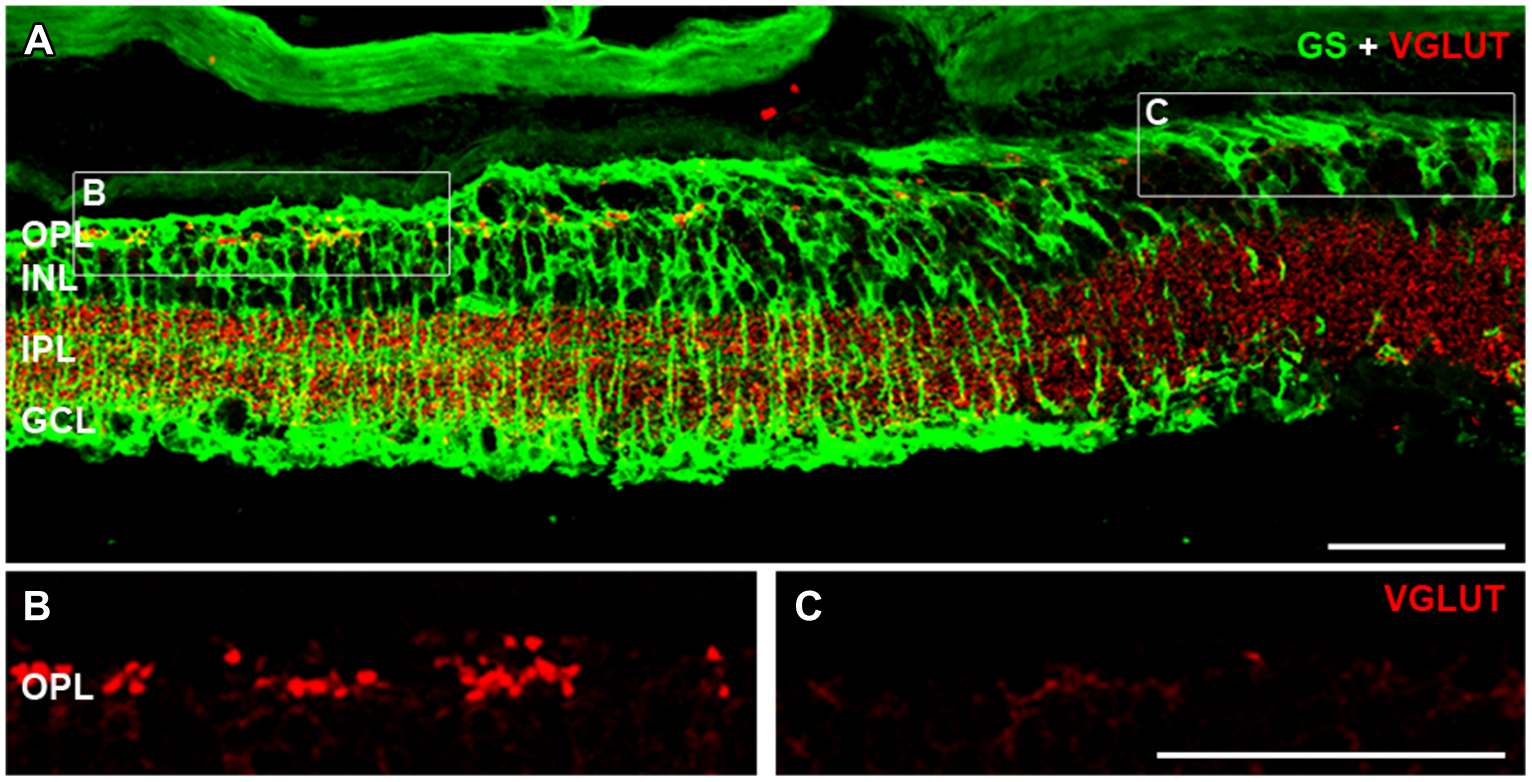
**Cone photoreceptor loss is evident in areas containing aberrant Müller cells.** Vertical sections of 6 month-old rd1-FTL retinae were double labeled for VGLUT1 (red) and GS (green) in order to investigate the presence of cone photoreceptor terminals in FTL-rich regions. VGLUT1 immunolabeling is expressed in the OPL and IPL **(A)**. High magnification of the OPL shows that in regions where GS is expressed, cone photoreceptor terminals remain **(B)**, however, in areas of low to no GS **(C)**, VGLUT expression is absent. Abbreviations: OPL, outer plexiform layer; INL, inner nuclear layer; IPL, inner plexiform layer; GCL, ganglion cell layer. Scale bar: 50 μm.

At late stages of retinal degeneration, phase 3 remodeling is characterized by marked anatomical changes that are thought to be precipitated by the loss of cone photoreceptor terminals. The association of cone-mediated remodeling on the patches of glial abnormality and FTL expression was assessed in the rd1-FTL (*n* ≥ 6 animals). **Figure 7** shows representative vertical sections taken from 6 month-old animals that were double labeled with GS and vesicular glutamate transporter (VGLUT1) a marker for the glutamate transporter within glutamatergic terminals (Sherry et al., 2003). Regions that showed GS labeling (**Figure 7A**) also displayed VGLUT1 labeling (**Figure 7B**), whereas areas that had low levels of GS immunolabelling, corresponded to regions that showed a complete loss of photoreceptor terminals as indicated by an absence of VGLUT1 labeling (**Figure 7C**). This finding was confirmed with labeling for the cone marker, peanut agglutinin, with normal GS regions exhibiting remnants of cone terminals, whereas GS depleted regions showed no labeling of synaptic terminals (data not shown). Overall, the results of this study highlight that during late stage retinal degeneration there are localized changes in inner retinal neurons (amacrine and ganglion cells) that are coincident with aberrant Müller cells and that these regions correspond to regions where cone terminals are lost.

## Discussion

*c-fos* is an intermediate early gene that is known to be regulated during cell death, altered neural function and neural plasticity. From approximately 4 months of age, a time where most photoreceptor nuclei have been lost, *c-fos* reporter (FTL) expression was increased in subsets of amacrine and ganglion cells across the central rd1-FTL retina. These regions of FTL expression were not correlated with cell death or inner retinal remodeling. These areas did correspond to discrete regions of abnormal Müller cells which showed reduced GS, Kir4.1, and Sox9 immunolabelling, in addition to aberrant cell cycle regulation. Furthermore, these aberrant Müller cells were present in regions that showed a complete loss of cone photoreceptor terminals. These results highlight that inner retinal changes occur in the rd1-FTL retina in a localized fashion and are associated with the loss of cone photoreceptor terminals and Müller cell anomalies.

### FTL Expression as a Marker of Inner Retinal Change

The results of this study show that *c-fos* reporter (FTL) expression changes in the rd1-FTL with age. Consistent with our previous findings (Greferath et al., 2009), retinal FTL expression was low at 3 months of age in the rd1-FTL compared to control FTL mice, most likely reflecting the lack of neural activity at this age. This is supported by previous work that indicates virtually all photoreceptor nuclei are lost in the central rd1 retina by 2 months of age (Jimenez et al., 1996). While FTL expression was initially low, it re-emerged within subpopulations of both amacrine and ganglion cells by 4 months of age (from P120). This increase in expression was not due to light-induced neuronal activation as we have previously reported in young (>P30) rd1-FTL animals (Greferath et al., 2004). Over the next 8 months (P120–P365) FTL expression increased within the central retina, yet never extended out into the peripheral retina. It must be highlighted that the oldest animals used in this study were 1 year of age and so peripheral FTL expression after this time cannot be discounted. The reason for this central localisation of *c-fos* reporter expression is unclear, however, a central to peripheral gradient in retinal degeneration has been reported in both the rd1 and rd1-FTL retinae (Carter-Dawson et al., 1978; Greferath et al., 2009).

The FTL transgene was used as a reporter for *c-fos*, an early intermediate gene that is known to be upregulated during cell death, neural function and neural plasticity. The increase in FTL expression in the amacrine and ganglion cells from 4 months of age was not associated with an increase in inner retinal cell death, as our results showed no difference in thickness of the INL, in either FTL-immunolabeled regions or neighboring FTL-negative regions. There was also no appreciable level of TUNEL labeling and certainly no specific localisation to FTL^+^ regions. Furthermore, the data did not support a role for *c-fos* reporter expression as a biomarker of inner retinal remodeling since anatomical remodeling was observed in FTL^+^ regions, as well as FTL^-^ regions. With previous work on the rd1 mouse model showing evidence of altered inner retinal neurochemistry, as well as aberrant amacrine and ganglion cell function (Stasheff, 2008; Chua et al., 2009; Choi et al., 2014), it is possible that these functional alterations are related to the observed FTL expression.

Our data showed that anomalies in glial cells, specifically Müller cells, coincide with the localized regions of FTL up-regulation. Based on the Sox9 labeling, Müller cells were reduced in number within FTL^+^ regions of retina. In addition, the Müller cells that remained in these regions were gliotic and showed reduced glutamate synthetase and Kir4.1 immunolabelling. In view of the importance of both glutamate turnover and potassium siphoning in maintenance of neuronal function (Bringmann et al., 2006), we propose that the Müller cell dysfunction observed in localized regions could play a role in the up-regulation of FTL expression within the inner retinal amacrine and ganglion cells. In support of this, Müller cells are known to directly modulate ganglion and amacrine cell function (Newman and Zahs, 1998; Miller, 2004). In addition, these aberrant Müller cells within the FTL expressing regions may be sites of future glial scar/seal formation which is known to occur in phase 2/3 remodeling (Marc et al., 2003). Previous work has indicated that, at time periods equivalent to the initiation of FTL expression (>P101), Müller cells exhibit distinct remodeling events, which contribute to the glial seal (Chua et al., 2013). Obviously further work will be required to determine whether these aberrant Müller cells modify the activity of their surrounding amacrine/ganglion cells, and serve as sites for future glial seals.

### The Role of Cone Photoreceptors in FTL Up-Regulation

While the majority of cone photoreceptors are lost by P90 – 100 in the rd1 mouse retina, cone remnants, including the presence of synaptic ribbons can remain for extended periods (Carter-Dawson et al., 1978). Our data showed that areas exhibiting dysfunctional Müller cells corresponded to regions without cone photoreceptor terminals. These data could reflect the importance of residual cone terminals in glial cell function, with the ultimate loss of these terminals resulting in glial dysfunction and/or FTL up-regulation within the inner retina. This idea is supported by work showing that cone loss occurs in patches (Jones et al., 2003), similar to the pattern of observed glial changes in the current study. Furthermore, previous work has shown that the preservation of cones can preserve the inner neural retina from significantly remodeling (Marc et al., 2003, 2007; Jones et al., 2012).

### Significance of Findings for Photoreceptor Restorative Therapies

Many studies of the rd1 mouse retina have shown that there are structural and functional changes in inner retinal cells following photoreceptor loss (Strettoi and Pignatelli, 2000; Strettoi et al., 2002; Marc et al., 2007; Margolis et al., 2008; Sekirnjak et al., 2011). Bipolar and horizontal cells change morphology and retract their dendrites (Strettoi and Pignatelli, 2000; Strettoi et al., 2002) as well as sprouting thin processes from their somata (Strettoi and Pignatelli, 2000). Ganglion cells have shown changes in their oscillatory activity (Margolis et al., 2008; Stasheff, 2008; Stasheff et al., 2011) and ion permeability (Marc et al., 2007; Chua et al., 2009). A caveat of many of these studies is that while they use animals at a stage when the majority of photoreceptors have died, they are in the early stages of the remodeling process (generally <P100). Evidence from this study suggests that the FTL model may provide an early biomarker for glial scar formation and the loss of remnant cones at later stages in the retinal degeneration process, thus allowing these processes to be explored. In particular, it may be useful in identifying and targeting retinal regions that are more amenable to the implantation of vision restorative therapies.

In summary, the results of this study highlight that the rd1-FTL mouse may be a useful and unique tool for analysing retinal integrity during late stages of retinal degeneration. The strong up-regulation of *c-fos* reporter (FTL) that occurred well after photoreceptor loss and remained in the central retina throughout later stages of retinal degeneration, were independent of cell death, structural remodeling and any residual light response. Rather, it was associated with areas of dysfunctional glial cells and complete cone photoreceptor loss. Inhibiting these glial changes or preserving cone photoreceptors may assist with maintaining inner retinal integrity.

## Conflict of Interest Statement

The authors declare that the research was conducted in the absence of any commercial or financial relationships that could be construed as a potential conflict of interest.

## References

[B1] Ali A. E. Wilson Y. M. Murphy M. (2009). A single exposure to an enriched environment stimulates the activation of discrete neuronal populations in the brain of the fos-tau-lacZ mouse. *Neurobiol. Learn. Mem.* 92 381–390. 10.1016/j.nlm.2009.05.004 19450699

[B2] Ali A. E. Wilson Y. M. Murphy M. (2012). Identification of neurons specifically activated after recall of context fear conditioning. *Neurobiol. Learn. Mem.* 98 139–147. 10.1016/j.nlm.2012.07.004 22820091

[B3] Bringmann A. Iandiev I. Pannicke T. Wurm A. Hollborn M. Wiedemann P. (2009). Cellular signaling and factors involved in Muller cell gliosis: neuroprotective and detrimental effects. *Prog. Retin. Eye Res.* 28 423–451. 10.1016/j.preteyeres.2009.07.001 19660572

[B4] Bringmann A. Pannicke T. Grosche J. Francke M. Wiedemann P. Skatchkov S. N. (2006). Muller cells in the healthy and diseased retina. *Prog. Retin. Eye Res.* 25 397–424. 10.1016/j.preteyeres.2006.05.003 16839797

[B5] Busskamp V. Duebel J. Balya D. Fradot M. Viney T. J. Siegert S. (2008). Genetic reactivation of cone photoreceptors restores visual responses in retinitis pigmentosa. *Science* 329 413–417. 10.1126/science.1190897 20576849

[B6] Carter-Dawson L. D. Lavail M. M. Sidman R. L. (1978). Differential effect of the rd mutation on rods and cones in the mouse retina. *Invest. Ophthalmol. Vis. Sci.* 17 489–498. 659071

[B7] Choi H. Zhang L. Cembrowski M. S. Sabottke C. F. Markowitz A. L. Butts D. A. (2014). Intrinsic bursting of AII amacrine cells underlies oscillations in the rd1 mouse retina. *J. Neurophysiol.* 112 1491–1504. 10.1152/jn.00437.2014 25008417 PMC4137253

[B8] Chua J. Fletcher E. L. Kalloniatis M. (2009). Functional remodeling of glutamate receptors by inner retinal neurons occurs from an early stage of retinal degeneration. *J. Comp. Neurol.* 514 473–491. 10.1002/cne.22029 19350664

[B9] Chua J. Nivison-Smith L. Fletcher E. L. Trenholm S. Awatramani G. B. Kalloniatis M. (2013). Early remodeling of Muller cells in the rd/rd mouse model of retinal dystrophy. *J. Comp. Neurol.* 521 2439–2453. 10.1002/cne.23307 23348616

[B10] Downie L. E. Hatzopoulos K. M. Pianta M. J. Vingrys A. J. Wilkinson-Berka J. L. Kalloniatis M. (2010). Angiotensin type-1 receptor inhibition is neuroprotective to amacrine cells in a rat model of retinopathy of prematurity. *J. Comp. Neurol.* 518 41–63. 10.1002/cne.22205 19882719

[B11] Farber D. B. (1995). From mice to men: the cyclic GMP phosphodiesterase gene in vision and disease. The Proctor Lecture. *Invest. Ophthalmol. Vis. Sci.* 36 263–275. 7843898

[B12] Fletcher E. L. Hack I. Brandstatter J. H. Wassle H. (2000). Synaptic localization of NMDA receptor subunits in the rat retina. *J. Comp. Neurol.* 420 98–112. 10.1002/(SICI)1096-9861(20000424)420:1<98::AID-CNE7>3.0.CO;2-U 10745222

[B13] Fletcher E. L. Jobling A. I. Vessey K. A. Luu C. Guymer R. H. Baird P. N. (2011). Animal models of retinal disease. *Prog. Mol. Biol. Transl. Sci.* 100 211–286. 10.1016/B978-0-12-384878-9.00006-6 21377628

[B14] Fletcher E. L. Kalloniatis M. (1996). Neurochemical architecture of the normal and degenerating rat retina. *J. Comp. Neurol.* 376 343–360. 10.1002/(SICI)1096-9861(19961216)376:3<343::AID-CNE1>3.0.CO;2-2 8956104

[B15] Fletcher E. L. Kalloniatis M. (1997). Neurochemical development of the degenerating rat retina. *J. Comp. Neurol.* 388 1–22. 10.1002/(SICI)1096-9861(19971110)388:1<1::AID-CNE1>3.0.CO;2-5 9364235

[B16] Gibson R. Fletcher E. L. Vingrys A. J. Zhu Y. Vessey K. A. Kalloniatis M. (2013). Functional and neurochemical development in the normal and degenerating mouse retina. *J. Comp. Neurol.* 521 1251–1267. 10.1002/cne.23284 23238927

[B17] Greferath U. Goh H. C. Chua P. Y. Astrand E. O’brien E. E. Fletcher E. L. (2009). Mapping retinal degeneration and loss-of-function in Rd-FTL mice. *Invest. Ophthalmol. Vis. Sci.* 50 5955–5964. 10.1167/iovs.09-3916 19661224

[B18] Greferath U. Nag N. Zele A. J. Bui B. V. Wilson Y. Vingrys A. J. (2004). Fos-tau-LacZ mice expose light-activated pathways in the visual system. *Neuroimage* 23 1027–1038. 10.1016/j.neuroimage.2004.06.044 15528103

[B19] Jimenez A. J. Garcia-Fernandez J. M. Gonzalez B. Foster R. G. (1996). The spatio-temporal pattern of photoreceptor degeneration in the aged rd/rd mouse retina. *Cell Tissue Res.* 284 193–202. 10.1007/s004410050579 8625386

[B20] Jones B. W. Kondo M. Terasaki H. Lin Y. Mccall M. Marc R. E. (2012). Retinal remodeling. *Jpn. J. Ophthalmol.* 56 289–306. 10.1007/s10384-012-0147-2 22644448 PMC3726038

[B21] Jones B. W. Marc R. E. (2005). Retinal remodeling during retinal degeneration. *Exp. Eye Res.* 81 123–137. 10.1016/j.exer.2005.03.006 15916760

[B22] Jones B. W. Watt C. B. Frederick J. M. Baehr W. Chen C. K. Levine E. M. (2003). Retinal remodeling triggered by photoreceptor degenerations. *J. Comp. Neurol.* 464 1–16. 10.1002/cne.10703 12866125

[B23] Kofuji P. Ceelen P. Zahs K. R. Surbeck L. W. Lester H. A. Newman E. A. (2000). Genetic inactivation of an inwardly rectifying potassium channel (Kir4.1 subunit) in mice: phenotypic impact in retina. *J. Neurosci.* 20 5733–5740. 10908613 10.1523/JNEUROSCI.20-15-05733.2000 PMC2410027

[B24] Lehre K. P. Davanger S. Danbolt N. C. (1997). Localization of the glutamate transporter protein GLAST in rat retina. *Brain Res.* 744 129–137. 10.1016/S0006-8993(96)01022-0 9030421

[B25] Marc R. E. Jones B. W. Anderson J. R. Kinard K. Marshak D. W. Wilson J. H. (2007). Neural reprogramming in retinal degeneration. *Invest. Ophthalmol. Vis. Sci.* 48 3364–3371. 10.1167/iovs.07-0032 17591910 PMC2408857

[B26] Marc R. E. Jones B. W. Watt C. B. Strettoi E. (2003). Neural remodeling in retinal degeneration. *Prog. Retin. Eye Res.* 22 607–655. 10.1016/S1350-9462(03)00039-9 12892644

[B27] Margolis D. J. Newkirk G. Euler T. Detwiler P. B. (2008). Functional stability of retinal ganglion cells after degeneration-induced changes in synaptic input. *J. Neurosci.* 28 6526–6536. 10.1523/JNEUROSCI.1533-08.2008 18562624 PMC3050548

[B28] Marti A. Jehn B. Costello E. Keon N. Ke G. Martin F. (1994). Protein kinase A and AP-1 (c-Fos/JunD) are induced during apoptosis of mouse mammary epithelial cells. *Oncogene* 9 1213–1223. 8134124

[B29] Mattapallil M. J. Wawrousek E. F. Chan C. C. Zhao H. Roychoudhury J. Ferguson T. A. (2012). The Rd8 mutation of the Crb1 gene is present in vendor lines of C57BL/6N mice and embryonic stem cells, and confounds ocular induced mutant phenotypes. *Invest. Ophthalmol. Vis. Sci.* 53 2921–2927. 10.1167/iovs.12-9662 22447858 PMC3376073

[B30] McLaughlin M. E. Sandberg M. A. Berson E. L. Dryja T. P. (1993). Recessive mutations in the gene encoding the beta-subunit of rod phosphodiesterase in patients with retinitis pigmentosa. *Nat. Genet.* 4 130–134. 10.1038/ng0693-130 8394174

[B31] Miller R. F. (2004). D-Serine as a glial modulator of nerve cells. *Glia* 47 275–283. 10.1002/glia.20073 15252817

[B32] Newman E. A. Zahs K. R. (1998). Modulation of neuronal activity by glial cells in the retina. *J. Neurosci.* 18 4022–4028. 9592083 10.1523/JNEUROSCI.18-11-04022.1998 PMC2904245

[B33] Niblock M. M. Lohr K. M. Nixon M. Barnes C. Schaudies M. Murphy M. (2012). Cells in the female retrotrapezoid region upregulate c-fos in response to 10%, but not 5%, carbon dioxide. *Brain Res.* 1433 62–68. 10.1016/j.brainres.2011.11.015 22137562

[B34] O’Brien E. E. Greferath U. Vessey K. A. Jobling A. I. Fletcher E. L. (2012). Electronic restoration of vision in those with photoreceptor degenerations. *Clin. Exp. Optom.* 95 473–483. 10.1111/j.1444-0938.2012.00783.x 22823954

[B35] Pittler S. J. Baehr W. (1991). Identification of a nonsense mutation in the rod photoreceptor cGMP phosphodiesterase beta-subunit gene of the rd mouse. *Proc. Natl. Acad. Sci. U.S.A.* 88 8322–8326. 10.1073/pnas.88.19.8322 1656438 PMC52500

[B36] Rauen T. Taylor W. R. Kuhlbrodt K. Wiessner M. (1998). High-affinity glutamate transporters in the rat retina: a major role of the glial glutamate transporter GLAST-1 in transmitter clearance. *Cell Tissue Res.* 291 19–31. 10.1007/s004410050976 9394040

[B37] Rich K. A. Zhan Y. Blanks J. C. (1997). Aberrant expression of c-Fos accompanies photoreceptor cell death in the rd mouse. *J. Neurobiol.* 32 593–612. 10.1002/(SICI)1097-4695(19970605)32:6<593::AID-NEU5>3.0.CO;2-V 9183740

[B38] Sekirnjak C. Jepson L. H. Hottowy P. Sher A. Dabrowski W. Litke A. M. (2011). Changes in physiological properties of rat ganglion cells during retinal degeneration. *J. Neurophysiol.* 105 2560–2571. 10.1152/jn.01061.2010 21389304 PMC3094174

[B39] Sherry D. M. Wang M. M. Bates J. Frishman L. J. (2003). Expression of vesicular glutamate transporter 1 in the mouse retina reveals temporal ordering in development of rod vs. cone and ON vs. OFF circuits. *J. Comp. Neurol.* 465 480–498. 10.1002/cne.10838 12975811

[B40] Stasheff S. F. (2008). Emergence of sustained spontaneous hyperactivity and temporary preservation of OFF responses in ganglion cells of the retinal degeneration (rd1) mouse. *J. Neurophysiol.* 99 1408–1421. 10.1152/jn.00144.2007 18216234

[B41] Stasheff S. F. Shankar M. Andrews M. P. (2011). Developmental time course distinguishes changes in spontaneous and light-evoked retinal ganglion cell activity in rd1 and rd10 mice. *J. Neurophysiol.* 105 3002–3009. 10.1152/jn.00704.2010 21389300

[B42] Strettoi E. Pignatelli V. (2000). Modifications of retinal neurons in a mouse model of retinitis pigmentosa. *Proc. Natl. Acad. Sci. U.S.A.* 97 11020–11025. 10.1073/pnas.190291097 10995468 PMC27141

[B43] Strettoi E. Pignatelli V. Rossi C. Porciatti V. Falsini B. (2003). Remodeling of second-order neurons in the retina of rd/rd mutant mice. *Vision Res.* 43 867–877. 10.1016/S0042-6989(02)00594-1 12668056

[B44] Strettoi E. Porciatti V. Falsini B. Pignatelli V. Rossi C. (2002). Morphological and functional abnormalities in the inner retina of the rd/rd mouse. *J. Neurosci.* 22 5492–5504. 12097501 10.1523/JNEUROSCI.22-13-05492.2002 PMC6758223

[B45] Vessey K. A. Fletcher E. L. (2012). Rod and cone pathway signalling is altered in the P2X7 receptor knock out mouse. *PLoS ONE* 7:e29990 10.1371/journal.pone.0029990 PMC3254638 22253851

[B46] Vessey K. A. Greferath U. Aplin F. P. Jobling A. I. Phipps J. A. Ho T. (2014). Adenosine triphosphate-induced photoreceptor death and retinal remodeling in rats. *J. Comp. Neurol.* 522 2928–2950. 10.1002/cne.23558 24639102 PMC4265795

[B47] Wilson Y. Nag N. Davern P. Oldfield B. J. Mckinley M. J. Greferath U. (2002). Visualization of functionally activated circuitry in the brain. *Proc. Natl. Acad. Sci. U.S.A.* 99 3252–3257. 10.1073/pnas.042701199 11867719 PMC122505

